# Dental Caries

**Published:** 1872-02

**Authors:** C. R. E. Koch


### ARTICLE II.

### *Dental Caries.*

BY C. R. E. KOCH, D. D. S.

Read before Illinois State Dental Society.

The name Dental Caries has, by many authors, been felt to be inadequate to convey a correct idea of the malady, and various other names, such as mortification, gangrene, and nigritis ossium have been substituted in its stead; none of these, however, being as appropriate as the first, all the late writers have adhered to it. As we cannot possibly expect to effect regeneration, it would hardly be judicious to attempt another christening, and perhaps we had better conclude with Shakspeare: "What's in a name? That what we call a rose, by any other name would smell as sweet."

Much has been written upon this subject from the earliest days of our profession, as a branch of medical science, up to the present day, by men to whose eminently scientific attainments and laborious researches we owe our present professional status. Yet it is truly astonishing to see what widely differing theories are advanced by nearly all of them. Thus nearly all the early English and American writers held that caries in bone and in teeth is identical, the latter as well as the former being caused by morbid action within the bony structure, and differing in appearance only, owing to the more densely constituted structure of the teeth, which prevents the pathological development and reparative functions as they occur in bone. Those authors who dissented from this view generally, admitted that if it was not already so caused, they were not prepared to deny that it might not sometimes be the result of morbid action within the dentinal substance. I will not stop here to recite all the different theories advanced, and will merely mention these as being the most prominent and around which all the others centre.

We may define Dental Caries as that pathological condition of the teeth, under which a portion of their structures become impaired, disintegrated and decomposed. Generally it attacks the dentine and enamel; and also, frequently the cementum at the necks of the teeth; and upon portions of the roots from which the gums and alveolus have been wasted. It most frequently is symmetrical in its occurrence, that is, it attacks the corresponding teeth of each side in a like locality and manner.

I must confess that I am not a believer in the doctrine that dental caries can ever commence from within the dentinal structure. Unless some corroding agent has first been conveyed to the dentine from without, through an original or accidental fissure in the enamel, caries never attacks it first. Hence I cannot see even an analogy to caries in bone; that the teeth may be, or may become, more or less susceptible to the action of corrosive agents, through vital or accidental causes, admits of no doubt. In so much, I am ready to admit that it is the result of chemico-vital action, that is, it *may be* vital in its remote or predisposing, and *it always is* chemical in its immediate or exciting causes.

Professor Garretson, in his recent eminent work on "Diseases and Surgery of the Mouth," thinks that these chemical agents do not act chemically upon the tooth substance, and have little to do in its dissolution, except as they act as irritants and produce inflammation, which, (although in consequence of difference of structure, differs in appearance in dentine and bone,) is in dentine as well as bone, the true exciting cause.

Prof. Bond, in his "Dental Medicine," says: "Caries of the teeth is a chemical erosion, by the action of the fluids of the mouth and the accidental matter dissolved in them, upon the salts of which the teeth are mainly composed." And, although he does not say that true caries is never found in teeth, yet he thinks that as generally met with in teeth, it is the result of chemical erosion, by acids upon the earthy matter.

The only vital action which does ever occur in dentine as caries progresses is an attempt to solidify the contents of the tubuli (be these liquor sanguinis, fibrillæ, or nerve fibres) by calcification as a means of defense; and thus the dentinal structure, to the extent of this calcification, becomes devitalized. Non-vitalized structures cannot perform vital functions, nor have vital diseases; and even should we, for argument sake, admit that enamel, and dentine having its fibrillæ or nerve fibres calcified, are vitalized structures, yet the fact that teeth which have had their pulps destroyed and removed, and which certainly cannot then be classed among the vital tissues, will be acted upon just the same as other teeth.

The vital theory is made utenable by the following facts: Caries frequently attacks enamel first, for which I believe the possession of vitality has never been claimed after the eruption of the teeth. It never commences within the substance of enamel or dentine. It never occurs before eruption of the teeth. If it were not caused by external, physical or chemical influences filling would be no remedy; but, upon the contrary, would tend to increase the difficulty, as it would, of necessity, increase the amount of inflammation. Human or animal teeth, used as artificial substitutes, become likewise affected. Caries never returns in a perfectly filled tooth. The cementum is never attacked by caries unless it is first denuded of its protecting gum and alveolus, and thus made subject to external agents. Filling teeth does not cause them to become carious on account of injury to the dentine by irritation and inflammation; on the contrary, crowded and ill-shaped teeth, judiciously filled, will be more likely to remain free from caries, on account of relief from the pressure of contiguous teeth, and the prevention of the accumulation of external corroding agents. All these reasons go far to prove the unstableness of the vital theory, and prove, pretty conclusively, that the cause must be looked for elsewhere.

The fact that even diluted acetic acid will readily dissolve the healthy and perfect enamel of the teeth, after extraction, in a short time, so that it can be scraped with the finger nail, ought to be taken in evidence that external agents do act upon the tooth substance.

Erosion, as described by Prof. Harris, is the effect of acid action upon the enamel near the cervical border. The dissolved particles, being removed by the fluids of the mouth, always leave a polished transverse groove. This being another species of caries, is another illustration of the effects of chemical action, as there certainly is no vital action in the surface of the enamel.

Many attempts have been made, by various authors to classify the different varieties of caries, but as the disease, in its exciting causes, is nearly, if not always, the same; and as the difference in appearance is owing principally to the particular agent causing it, the different degrees of density in the tooth, or the rapid or slow progress of the disease, any such attempt must prove futile.

Oftentimes caries makes its advance and destroys the whole coronal dentinal substance before the patient is aware of the fearful trespass that has been committed upon his premises. From this procedure of the destroying agent, it has been inferred that the beginning of the operation was from within the tooth substance. There is no doubt, however. that in all such cases, a fissure, either original or accidental, existed in the enamel through which the acrid fluids permeated, thus performing their work quietly before the enamel gave way and exposed the ravages committed; or, perhaps, the most excruciating of pains may perform the duty of monitor.

When caries attacks the dentine first, the enamel over the affected part generally at first takes a slightly opaque hue, which, as the disease progresses, grows darker, so that by the time the destruction approaches completion, it may present the appearance of a deep ash color. It is impossible, however, to give a correct description of its appearance, as this is in a great measure influenced by the color and texture of the teeth. Thus the pearly white teeth, which are softest, will most generally decay very rapidly, and present a blueish dark appearance, while yellow teeth, which are most dense, and hence give way to the attacks of the destroyer more slowly, will take a deeper yellow, brown or black shade.

Temporary constitutional disease may so affect the fluids of the mouth as to render them very acrid, and at the same time may so deplete the general vital functions as to render the teeeth more susceptible to the action of these fluids. Under these circumstances caries may advance rapidly. The general health being restored, and the secretions having again become nomal, the further progress of the disease may be arrested, and the already decomposed mass change color and assume the appearance of caries of slow progress.

Mr. Tomes holds that caries in the tooth is always heralded by slight uneasiness or pain from its incipiency. Particularly is this noticed by persons accustomed to bestow considerable attention upon their teeth.

When caries attacks teeth at their cervical line, and has penetrated the cementum, it is generally very sensitive, and I do not know that I can assign any good reason for this being more so in this case than in any other locality, as in any case the substance undergoing change is peripheral dentine. Oftentimes in excavating a carious cavity we find a *noli me tangere* at a point farthest removed from the pulp. We are very much indebted to the microsciope, as through its powers we have learned many things oblivious to us before, and I trust that it may yet succeed in explaining the pathology of sonsitive dentine.

You have, no doubt, all of you seen the theory advanced in the April number of the *Dental Cosmos* of the current volume, by a brother in Brazil, who suggests that this condition is owing to a variety of animalculæ called denticolæ, which luxuriate in the carious cavity, and when disturbed by the rude evolutions of the excavator, seek refuge in the open mouths of the dentinal tubuli, and thus displacing part of the soft solids contained therein, by pressure upon the pulp, produce pain. I do not propose to enter into any argument upon this theory pro or con, but would suggest that the microscope ought readily to decide whether or not there is anything tangible in such theories. The most rational theory no doubt is that caries reaches the peripheral end of one or more uncalcified fibrillæ or nerve fibres, its progress not having been opposed by the zone of consolidation, which convey readily any sensation produced by contact of an instrument, acrid secretions or thermal changes to the nervous centers.

The causes of caries, so far as observed are numerous. It would be almost impossible, in a paper like this, to be very exhaustive in their examination, As we have seen before, they may be divided into predisposing and exciting causes.

Let us first look at the predisposing causes, which may be either hereditary, congenital or accidental.

That children, partaking in a marked degree of the general physique of either of their parents, will have a similarity in shape, structure and arrangement of their parents teeth, I believe is generally conceded, and unless this parent has become subject to the ravages of syphillis, or other transmissable constitutional vice, after his or her teeth were formed, or some derangement of the child's own constitution during the formative process has occurred, to alter the transmitted good condition of this parent's teeth, this law holds good. In other words, feeble parents, and those possessing poor teeth, will never find that their progeny have particularly strong teeth; whereas, parents who possess the strongest character of teeth, may fail to transmit these.

By congenital, we do not merely understand those causes which were operative during intra-uterine existence of the child, but also those which are so previous to the eruption or birth of the tooth. This class of predisposing causes is subject to a variety of conditions, chief among which are imperfect nutrition supplied to the mother during pregnancy and lactation, and the child up to puberty, and lack of the assimilation, either through the digestive organs failing to appropriate from the blood the elements required, or the tissues failing to appropriate from the blood the elements essential to them, either in quantity or quality.

Diseased condition of the decidious teeth may, by interfering with the calcification of the germs of the permanent teeth, leave a baneful influence upon the latter.

Among the accidental causes predisposing to caries, we must count, first, everything that will give more ready access to the action of the corroding agents; and secondly, everything that will aid in the formation of these agents.

Blows fracturing enamel or dentine, crowded and illy arranged teeth, the presence of supernumerary teeth, sudden thermal changes, as from hot tea to ice water, badly performed dental operations, &c., may be cited as of the first class. Any prolonged general derangement of the system, fevers, indigestion, nervous excitements, carious or otherwise diseased teeth, &c., are instances of the latter class.

Pregnancy is generally followed and accompanied by rapid progress of caries in the teeth. This may be owing to the fact, that all the vital energies are engaged in a remote direction, and thus the approach of the caries is not resisted by the formation of the zone of consolidation (of Tomes) or of calcification; or it may be caused by the fact, that the secretions in this condition become surcharged with acrid agents. But, we know that this end is accomplished—no matter how—and the old maxim, "for every child a tooth," upon examination may not be as preposterous as it may at a first glance appear.

The exciting cause is always of a chemical nature, and is either owing to the presence of acid or alkali, but most generally acid. I must confess never to have found an alkaline reaction in caries when applying a test, but believe that this must have been owing more to an improper procedure on my part than to the actual condition of the caries tested. The variety of caries in which the white, chalky mass remains, and presents the appeatance of having lost all its organic particles, certainly would cause one to believe that alkali must have been its cause. Even dilute acids will readily act upon perfectly formed and healthy enamel; but unless the dentine has become exposed, and is of a highly organized description, alkali will not act upon the teeth.

But no matter whether the agent is acid or alkaline, it is either held in solution in the fluids of the mouth, as they are secreted from the salivary glands and mucous membrane, generated by fermentation from the particles of the food in the interstices of the teeth; or it may be introduced as food, or as medicine. Acid eructations from the stomach, as in dyspepsia, often vitiate the fluids of the mouth. Carious cavities in teeth, and the remnants of teeth which have long since passed away, often serve as vessels for fermentation of particles of food. These agents sometimes act primarily upon the enamel, but more generally they penetrate the sulci of the molars and bicuspids, in which the enamel is not perfectly united, or any other fissure or opening, and reaching the dentine, act quite readily.

Before looking at the remedies with which to combat the evil of dental caries, let us briefly consider the consequences it may produce. The immediate result is usually a most severe toothache, resulting frequently in inflammation and suppuration of the pulp; in periosteal inflammation, alveolar abscess and loss of the tooth. Facial neuralgia, hemiplegia, and other affections of the nervous system, are often caused by carious teeth. The diseased condition of the teeth prevents proper mastication, and this, together with their foul and decaying matter becoming mingled with the ingesta, is the frequent cause of dyspepsia. Consumption is also said to be incited by the fetor of the inhalations. Deafness and functional difficulties of other distant organs have been ascribed as the result of caries in teeth. Insanity has been cured by the extraction of carious teeth.

The remedies at our hand with which to combat this universal enemy ought now to receive at least a brief notice, and as prevention should always receive our first attention, let us see what can be done by way of prophylaxis.

Understanding the causes, it would be an easy matter to say, avoid these and you will avoid the disease. And it is right here that we can be of most service to our patients.

In the case of a hereditarily poor condition perhaps less can be done than in any other case, but even here much may be accomplished by strict attention to hygienic laws. First and foremost, it is of the utmost importance that the constitutional health is always kept "at par," and especially so during the period of formation. Parents should be taught that the material out of which teeth and bones are made must be furnished in the food. Soil that is only capable of raising corn, which contains very little of the bone-earth, will not produce wheat unless previously fertilized with lime, which base largely enters into the composition of the latter.

We ought to point out that pastry and sweetmeats will aid in the formation of neither "bone nor sinew;" and that sugar, starch and fats only supply the calorifacient elements of food, and that for the truly nutrient elements we must look elsewhere. Eggs, milk and lean of meat are prominent representatives of the latter class.

We should teach the folly of throwing away two-thirds of the nutriment contained in the grain of wheat in the preparation of our flour; especially during the period of growth of the bony structure. The lime salts can, however, be abundantlv supplied in other vegetable productions, nearly all the esculent roots containing them. Beans, cabbage, lentels, &c., and the pomaceous fruits, are also richly stocked with the phosphates. Hence it is by no means essential to become a Grahamite to practice supplying proper food for teeth and bones. The importance of this doctrine is particularly to be impressed upon mothers during pregnancy and lactation, as a lack of these ingredients in the food will certainly tell upon the mother's own teeth, if not upon those of the infant, as the latter may be nourished from the stores in the mother's frame.

It is always desirable to supply those elements of food in their organized form, but, as under certain conditions, the taste may become depraved, or very fastidious, we may substitute, instead of the products of nature's laboratory, those of the pharmacist, and supply the required elements in the shape of chemical foods, of which perhaps many formulas exist, and which rely upon combinations of the phosphates of lime, magnesia and iron for their efficacy. Whenever it is evident that nutrition is imperfect these phosphates are indicated.

If the elements contained in the food are not properly assimilated, of course the structures in process of formation must suffer. As this is very often the result of indigestion, it is essential that the cause be removed. Frequently carious teeth, by imperfectly performing the process of mastication, and by mixing with the food their integral and contained particles, seriously interfere with proper digestion. Such teeth should either be removed or restored to a healthy and serviceable condition.

The system should be generously treated with tonic, and fresh air exercise, especially to people of sedentary habits, ought to be encouraged. As food does not consist merely of tne ingesta—fresh air and the benign rays of the sun also being food—we ought not to disregard the importance of the latter. But perhaps all this would more properly belong to a paper on hygiene; therefore let us now proceed to consider direct remedial treatment.

First of all the teeth should be kept clean ; and to this end we should not rely merely upon our toooth-brush, nor should we trust too much to our dentifrice, which, no matter how efficient it may be, yet cannot in all cases and places be effective. After eating, a quill or orange wood toothpick should be used, and, if possible a soft piece of cloth or silk be passed between the teeth, and drawn backward and forward, and up and down.

The matter of tooth powders has received considerable attention at society meetings, and various formulas have been recommended. It is extremely doubtful, however, if any formula can be devised which would be just the thing in all cases. It should be free from chemical or mechanical irritants, and yet possess detersive powers. If the mouth be in an inflamed condition, astringents should be added. As in most mouths there is an acid reaction, *chalk* is probably the most proper base, as this will neutralize any free acids; and even in alkaline mouths it certainly can do no harm. It may be extremely doubtful whether it is judicious to add agents capable of neutralizing alkali to a tooth powder, as the difficulty of controlling and defining the action of such agents ought not to be overlooked.

Perhaps ordinarily, precipitated chalk used as a dentifrice and allowed to remain in the mouth until it is washed away, especially at night,—and after eating acid fruits, will be as effective as any topical prophylactic. However, as it is much safer to prevent the accumulation of deleterious agents than to make topical applications to neutralize them, we should impress upon our patients the necessity of using the mechanical means, at our hands, to this end.

Cracking nuts and drawing nails with the teeth are feats which should be discouraged.

Prof. Garretson, in the work before referred to, denies that any injury, beyond an irritant one, can be produced by sudden thermal changes. The climatic changes, (which have been assigned as causes for the great extent of caries in teeth in this country) cannot very well have any direct effect upon the tooth enamel. The climate of California is probably as even a one as that of any other country, yet I am still to be informed that caries occurs less there than in the States east of the Rocky Mountains. That enamel of the teeth is cracked or fractured, similarly to glass, by sudden changes of temperature, can easily be demonstrated. By first subjecting a perfectly enameled tooth to the spray of ether or rhigolene, and suddenly plunging it into boiling water, cracks or fissures will be produced in the enamel. This, of course, is too extreme a test to occur within the oral cavity; still, it is best to avoid great thermal differences in food.

Salivary calculus, although never a cause of caries, except as it acts as an irritant to the soft tissues, and thus vitiates the secretion, should be removed.

Crooked, or very irregularly arranged teeth, should be corrected, in such manner as the exigencies of the case may require, either by regulating, removal of one or more teeth, or judicious filing. All mechanical irritation should be removed; artificial dentures, if worn contiguous to natural teeth, should especially be kept clean. All remaining roots of wasted teeth should be removed. Frequently powerful acids are given as tonics by physicians, and, as patients do not always know the composition of medicines prescribed for them, it is best to advise that all medicines be taken through a glass tube, so that their contact with the teeth may be prevented.

Sometimes, when caries has actually commenced, we may, in highly vitalized teeth (that is those of very young persons, or those in which calcification has progressed very slowly) by removing and preventing the action of the exciting agents, and the dietetic and hygienic treatment, as above indicated, prevent the further progress, and cause the more ready calcification and strengthening of the dentine. Nature often takes a similar course, in cases where a marked improvement in the general health has arrested caries of rapid progress very suddenly, and, if it existed in a self-cleansing surface, finally.

When caries has progressed to some extent, the treatment can only be topical, and must be mechanical in its nature. If it does not penetrate far beyond the enamel in lateral cavities, it can often be removed by the file and other suitable instruments; and if this is done in such a manner as to leave a perfectly smooth and polished surface, and one easily cleansed, and the patient is taught to observe proper care, and complies with his instructions, it will never return.

If so situated as to preclude this remedy, or if the carious cavity is too extensive, all the decomposed and softened matter should be removed thoroughly, especially from the walls of the cavity and the marginal edges of the cavity, and this cavity thoroughly filled with the most suitable material.

Should there not be sufficient healthy tooth substance left to support a proper filling, the only remedy at our hands is cold steel, in the shape of forceps.

The treatment of caries complicated with exposure or disease of the pulp or periosteum scarcely belongs here, and

I have only considered simple caries, as any complication must first be reduced to a simple form before the mechanical remedy can be applied.

As caries always attacks weak parts first, we ought carefully to search out these, and if the condition of the mouth demands, we should boldly proceed to fortify these by throwing up defensive works wherever an attack seems imminent.

Wherever in children we find the sulci of the molars or bicuspid in an imperfect condition of enamel fusion, and especially in children of weak constitutions and with soft teeth, it ought to be considered good practice to cut out this imperfectly formed enamel, shape into a proper cavity, and fill the same with gold. In doing this we need not fear the accusation of making holes for the sake of obtaining a fee, as the intelligent will appreciate our efforts, and will be glad to reward them. In the case that our patients cannot esteem or appreciate this operation, we must content ourselves with the applause of our own consciences.

When the teeth are atrophied, that is, either pitted or grooved or having thin or imperfectly formed enamel upon their exposed or self-cleansing surfaces, it is not always so plain what course of treatment is most indicated. Oftentimes, when this is the result of interference during the formative process by disease, and not of hereditary transmission—the general system recovering—the soft enamel may gradually become worn away, leaving under it a firmly calcified dentine which is scarcely secondary to enamel itself in resisting power.

Perhaps, unless the pits or grooves are very marked and extend entirely to the dentine, it would be best not to interfere at once, but to give admonition to the patient to observe the before named general laws and to present him or herself for frequent examinations, and thus by watching these teeth we may arrest caries as soon as it may set in.

It is impossible, however, to lay down laws for practice in all cases, and the operator must draw upon his observation and reflective powers—in short use his brains—before determining what ought to be done in any given case.

