# Errata

**Published:** 1872-03

**Authors:** 


*Errata.*—In Dr. C. R. E. Koch's article on "Dental Caries," in the February number of Journal, on page 439 fifth line from top, read "that which" in place of "that what," and on page 444 ninth line from bottom the word "blood" should be "food."

